# Notch1 phenotype and clinical stage progression in non-small cell lung cancer

**DOI:** 10.1186/s13045-014-0104-2

**Published:** 2015-02-06

**Authors:** Dat Nguyen, Larry Rubinstein, Naoko Takebe, Lucio Miele, Joseph E Tomaszewski, Percy Ivy, James H Doroshow, Sherry X Yang

**Affiliations:** National Clinical Target Validation Laboratory, Division of Cancer Treatment and Diagnosis, National Cancer Institute, National Institutes of Health, Bethesda, MD 20892 USA; Biometric Research Branch, Division of Cancer Treatment and Diagnosis, National Cancer Institute, National Institutes of Health, Bethesda, MD 20892 USA; Cancer Therapy Evaluation Program, Division of Cancer Treatment and Diagnosis, National Cancer Institute, National Institutes of Health, Bethesda, MD 20892 USA; Stanley Scott Cancer Center, Louisiana State University Health Sciences Center and Louisiana Cancer Research Consortium, New Orleans, LA USA; Division of Cancer Treatment and Diagnosis, National Cancer Institute, National Institutes of Health, Bethesda, MD 20892 USA

**Keywords:** Lung cancer, N1-ICD, Notch1, NSCLC, Stage

## Abstract

**Background:**

Notch1 transmembrane receptor is activated through ligand-binding- triggered proteolytic cleavages and, upon release, the intracellular domain (N1-ICD) translocates into the nucleus and modulates target gene transcriptions. Notch activation has been implicated in tumorigenesis in an increasing number of human malignancies including non-small cell lung cancer (NSCLC). However, Notch1 in distinct expression patterns and activation status with tumor progression remains to be defined in NSCLC.

**Methods:**

Notch1 and activated Notch1, N1-ICD, were examined by immunohistochemistry in 58 cases of stage I to IV NSCLC tumors. Association between Notch1 or N1-ICD expression and clinicopathological factors was assessed via correlation coefficient r statistics. *P*-values are two-sided.

**Results:**

Detectable tumor Notch1, predominantly localized to the membrane and cytoplasm, was observed in 29 cases (50%, 95% Blyth-Still-Casella confidence interval 37 – 63%). It was negatively associated with stage (r = - 0.43, *P* < 0.001) and nodal status (r = - 0.33, *P* = 0.01), but not tumor size. In contrast, nuclear N1-ICD expression level was low and found in 12% of NSCLC patients, neither significantly associated with stage nor nodal status. Upon Notch1 activation in vitro, a mostly extra-nuclear staining was substantially turned into the nuclear signal in cancer cells.

**Conclusions:**

Notch1 in the largely inactivated phenotype is inversely associated with clinical stage progression in NSCLC. Notch1, rather than activated N1-ICD, may be a context-dependent restrictive factor to nodal metastasis.

## Background

Lung cancer is the leading cause of cancer-related mortality in men and women in the United States and Europe [1]. Approximately 85% of all lung cancers are non-small cell lung cancer (NSCLC), which includes squamous cell carcinoma, adenocarcinoma and large cell carcinoma (LCLC). The Notch signaling pathway is a highly evolutionally conserved signal transduction network that is critical for cell fate specification in a context-dependent manner during and after development in various organ tissues [2,3]. Notch signaling is frequently deregulated in human hematological malignancies [4,5] and solid tumors including NSCLC, through gene mutations and aberrant expression of Notch receptors [6,7]. Specifically, Notch signaling maintains a balance between cell proliferation and apoptosis and has been shown to be oncogenic or tumor-suppressive depending on the cancer types; it can be both oncogenic and tumor-suppressive within one cancer type such as in B-cell malignancies [8]. Activated Notch1, in co-operation with Myc or through regulation of expression of epidermal growth factor receptor (EGFR), was implicated in the tumorigenesis, proliferation and survival of NSCLC models in preclinical studies [9,10]. Moreover, hypoxia via HIF1α stabilizes and activates Notch1 in lung adenocarcinomas. In turn, Notch1 activates the IGF-1R pathway, promoting cancer cell survival under hypoxia [11,12]. Inhibition of ADAM-17 — a critical step of ligand-dependent activation of Notch signaling led to substantial cell death and reduced tumorigenesis in NSCLC cell lines and xenograft tumor models. Activated Notch1 was associated with poor survival in NSCLC patients without p53 mutations [13]. Despite the substantial data of Notch1 in tumorigenesis and tumor cell survival, association of Notch1 expression levels and patterns with tumor progression in terms of tumor size and metastasis remains to be delineated.

The Notch receptors are non-covalently bound heterodimeric proteins, consisting of a large N-terminal extracellular portion (N^EC^) featuring multiple EGF-like repeats and the Notch transmembrane domain (N^TM^), which includes an extracellular stub, transmembrane segment and intracellular domain (NICD) [14,15]. The NICD, upon sequential cleavages of N^TM^ by ADAM10 or 17 and gamma-secretase, is released from the plasma membrane and translocates into the nucleus, where it activates target gene transcriptions. In addition, the EGF repeats in the N^EC^ is calcium-dependent. Calcium depletion by EDTA can destabilize N^EC^ and lead to its dissociation from N^TM^, activating the signaling of some of the Notch receptors such as Notch1 and 2 [16].

Agents targeting the Notch pathway including γ-secretase inhibitors (GSIs) and monoclonal antibodies (mAbs) to Notch ligands and Notch receptors are currently in early clinical development across a range of advanced human malignancies. GSIs have numerous possible targets, but their anti-neoplastic effects are thought to be due mostly to Notch inhibition, primarily Notch1, observed by several studies [15,17,18]. However, single-agent antitumor activity of Notch inhibitors, as determined by radiologic responses, was observed in only about 3% of patients treated with a Notch pathway inhibitor according to available clinical trial data [15]. Unexpectedly, clinical activity has not been observed in patients whose tumors harbor Notch1 mutations thus far [19,20]. Moreover, systemic Notch inhibitor administration causes significant toxicities with dose limiting gastrointestinal adverse events (goblet cell metaplasia and secretory diarrhea) [15].

Given the current clinical challenges and specific features of the Notch signaling, it is important to characterize expression levels and patterns in association with activated or inactivated Notch1, and in turn elucidate the target status quo for therapeutic intervention. In this study, we examined Notch1expression in NSCLC, and identified those tumors with abundant Notch1 as well as activated Notch1, N1-ICD, at γ-secretase cleavage site (N1-ICD-V1754). Notch1 and N1-ICD-V1754 expression patterns were characterized in cancer cell lines via modulation using EDTA and in human NSCLC tumors. Particularly, this investigation tested the hypothesis that inactivated Notch1 may play a suppressive role in tumor progression through defining Notch1 or N1-ICD-V1754 status in relation to clinicopathological factors such as tumor differentiation, nodal status and clinical stage.

## Results

### Modulation of Notch1 expression patterns following activation by EDTA in cancer cells, and validation of antibodies

**Figure 1:**
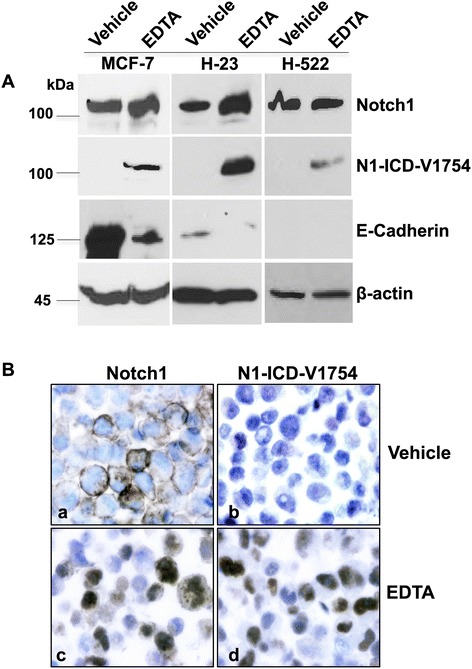
**Expression of Notch1, N1-ICD-V1754 and E-cadherin by Western blot and immunocytochemistry in cancer cells.** Western blot analysis demonstrates specific bands of the respective proteins of Notch1, N1-ICD-V1754 and E-cadherin in MCF-7, NCI-H23 and NCI-H522 cells **(A)**. Notch1 (a, c) and N1-ICD-V1754 (b, d) expression in formalin-fixed and paraffin-embedded NCI-H23 cells **(B)**. Magnifications x600.

To characterize Notch1 expression patterns, NSCLC and breast cancer cells were treated with EDTA to activate Notch1. With and without EDTA exposure, EP1238Y antibody identified Notch1 protein of ~125 kDa, corresponding to the approximate size of N^TM^ of Notch1 (Figure 1A); D3B8 antibody detected N1-ICD-V1754 of ~110 kDa in NSCLC NCI-H23, NCI-H522 and breast cancer MCF-7 cells after EDTA treatment. Immunocytochemistry analysis revealed that Notch1 was mainly bound to the membrane and cytoplasm, and upon EDTA treatment, N1-ICD-V1754 was rapidly induced and translocated to the nucleus in NCI-H23 cells (Figure 1B). These results indicate specific modulation of Notch1 expression patterns upon Notch1 activation by EDTA, switching a mostly membranous/cytoplasmic staining to the dark-brown signal in the nucleus in NSCLC cells expressing Notch1. In addition, specificity and utility of the two antibodies were validated for use in paraffin-embedded sections. Further, the cell adhesion molecule E-cadherin was decreased in MCF-7 and NCI-H23 cells (Figure 1A).

**Figure 2:**
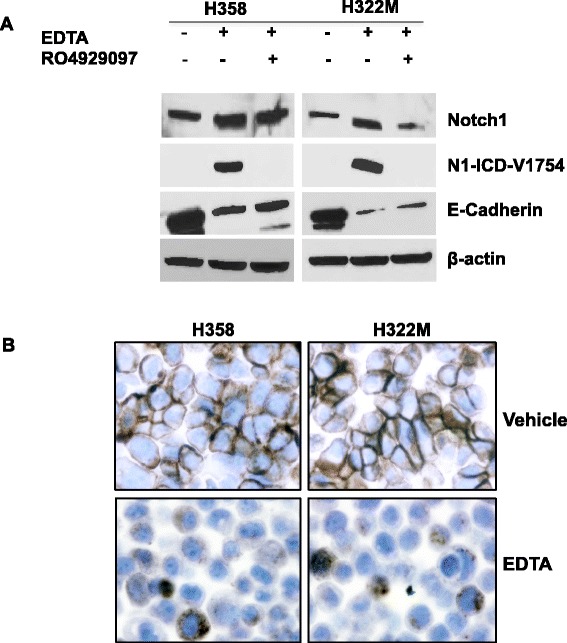
**Confirmation of expression of Notch1, N1-ICD-V1754 and E-cadherin in additional NSCLC cells.** Expression of Notch1, N1-ICD-V1754 and E-cadherin were analyzed using Western blot in NSCLC NCI-H358 and NCI-H322M cells **(A)**. E-cadherin expression was examined by immunohistochemistry in NSCLC NCI-H358 and NCI H322M cell lines **(B)**. Magnifications x600.

To ascertain and extend the findings, NCI-H358 and NCI-H322M NSCLC cells were also subjected to EDTA treatment. As expected, N1-ICD-V1754 was markedly induced in these cells that express Notch1 (Figure 2A). The reduction of E-cadherin by EDTA treatment was also studied further with these two cell lines. It was again noted that appearance of N1-ICD-V1754 was coupled with a significant reduction in E-cadherin expression by both Western blot and immunocytochemistry (Figure 2A and B). Treatment with EDTA in the presence of RO4929097 prevented Notch1 activation and partially rescued E-cadherin from the decrease (Figure 2A).

### Expression of Notch1 and N1-ICD-V1754 in human NSCLC

**Figure 3:**
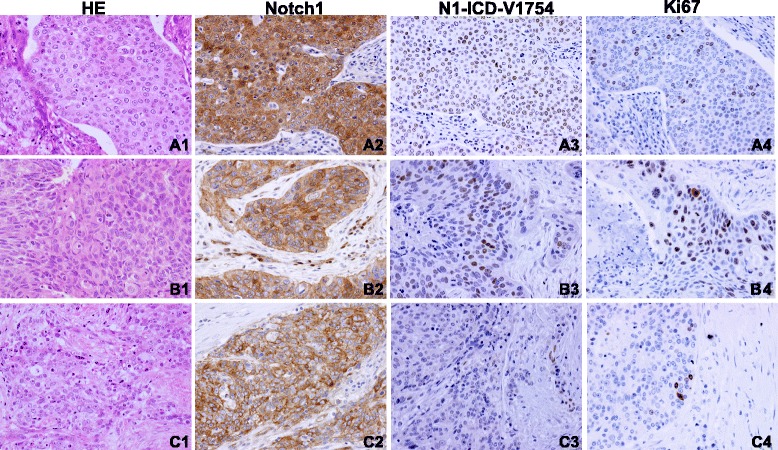
**Representative staining patterns of Notch1, N1-ICD-V1754 and heterogeneous tumor cell proliferation (Ki67) in NSCLC tumor tissues.** Notch1, N1-ICD-V1754 and Ki67 in a case of undifferentiated carcinoma of the lung, showing high level of membranous/cytoplasmic/nuclear Notch1 expression **(A2)**, nuclear N1-ICD-V1754 **(A3)**, and Ki67 labeling **(A4)**. Notch1, N1-ICD-V1754 and Ki67 in a case of squamous cell carcinoma of the lung, displaying high level of cytoplasmic/membranous/nuclear Notch1staining **(B2)**, heterogeneous N1-ICD-V1754 **(B3)**, and Ki67 proliferation **(B4)**. Notch1, N1-ICD-V1754 and Ki67 in a case of adenocarcinoma of the lung, exhibiting cytoplasmic/membranous Notch1 signal **(C2)** and some nuclear N1-ICD-V1754 staining on the right **(C3)**, and Ki67 expression **(C4)**. HE staining of undifferentiated carcinoma, squamous cell carcinoma and adenocarcinoma of the lung **(A1**, **B1**, and **C1)**. Magnifications x200.

All levels of tumor Notch1expression were found in 29 of the 58 NSCLC cases (50%, 95% CI 37% – 63%), with 8 cases (14%) exhibiting strong staining. These included 5 of the 35 cases of squamous cell carcinomas, 2/19 adenocarcinomas, and 1/4 undifferentiated or large cell undifferentiated carcinomas (Table 1; Figure 3). Notch1expression was not statistically different between adenocarcinomas and squamous cell carcinomas (P = 0.57; Table 1). Similar to the expression pattern observed in cancer cells, membranous/cytoplasmic/nuclear Notch1, with an extra-nuclear staining as the major signal, was observed in NSCLC tumors. In addition, it was high levels of Notch1 expression, 6 out of 8, which were coupled to heterogeneous N1-ICD-V1754 expression (Figure 3).

**Table 1:** **Notch1 and high Notch1expression in NSCLC**

**Histology**	**No. of samples**	**Expression**		**High expression**	
		**No. of positive**	% (95% CI)*	**No. of positive**	**% (95% CI)**
**NSCLC**	58	29	50 (37 – 63)	8	14 (6 - 25)
**Squamous cell carcinoma**	35	19	54 (37 - 71)	5	14 (6 - 29)
**Adenocarcinoma**	19	8	42 (22 - 66)	2	11 (2 - 32)
**Undifferentiated/large cell carcinoma** ***P*** **value** ^**†**^	4	3	75 (25 - 99)	1	25 (1 - 75)
**Squamous cell vs. adenocarcinoma**			0.57		1.0

*Blythe-Still-Casella 95% confidence interval (CI).

^†^Comparison of percent positivity by Fisher’s Exact Test; 2-sided *P* value.

Expression of N1-ICD-V1754 was exclusively nuclear and detected in 7 of the 58 cases (12%, 95% CI 6-22%). The seven were all from 29 Notch1-positive cases, and none from Notch1-negative ones. Among those, the expression levels were low in 6 cases and intermediate in one sample, relative to NCI-H23 cells treated with EDTA. In the context of heterogeneous Notch1 activation, we observed a heterogeneous Ki67 labeling/tumor cell proliferation (Figure 3).

### Association between Notch1 or N1-ICD-V1754 and clinicopathological covariates in NSCLC

Analysis of disease-related clinicopathological parameters showed neither a significant association between Notch1 expression and demographics including age and sex, nor a correlation between Notch1 and tumor grade or tumor size (Table 2). However, there was a significant negative association between Notch1 and clinical stage (r = - 0.43; *P* < 0.001) or between Notch1 and nodal status (r = - 0.33; *P* = 0.01). In contrast, N1-ICD-V1754 was not significantly associated with stage (r = - 0.06; *P* = 0.65) and nodal status (r = - 0.09; *P* = 0.50).

**Table 2:** **Association of Notch1with clinicopathological variables in NSCLC**

**Variable**	**No. of cases**	**Correlation co-efficient, r**	***P*** value*
**Age**	58	0.02	0.91
**Sex**	58	0.15	0.25
**Grade**	45	-0.07	0.66
**Tumor size**	57	-0.04	0.76
**Nodal status**	56	-0.33	0.01
**Stage**	57	-0.43	≤0.001

*By correlation coefficient r statistics, two sided *P* value.

## Discussion

Our data demonstrate that all levels of Notch1are detected in 50% of stage I to IV NSCLC tumors, and it is predominantly localized in the cell membrane and cytoplasm. Notch1 is largely inactivated in NSCLC as only a small fraction of NSCLC samples heterogeneously express low levels of N1-ICD-V1754 [21]. Immunocytochemistry results clearly show that Notch1 translocation from the cell membrane to the nucleus upon activation by EDTA treatment, indicative of the function of the canonical Notch signaling in NSCLC and breast cancer cells [15,16,22]. EDTA treatment could elicit a degree of Notch1 activation comparable to that resulted from Notch ligand Delta-1 exposure [16]. Pre-treatment with and in the presence of RO4929097 during EDTA treatment abolishes Notch1 activation, suggesting that Notch1 activation mediated by EDTA is gamma-secretase-dependent in NSCLC cells.

Importantly, the inactivated Notch1 configuration in NSCLC is inversely associated with locoregional node metastasis, whereas it is not significantly associated with tumor size. Thus, the negative association between Notch1phenotype and clinical stage progression is likely driven by nodal status. The findings support our hypothesis that inactivated Notch1 may serve as a context-dependent restrictive factor of tumor cells to local-regional spread. The scenarios that inactivated Notch1 is protective against nodal spread could be explained as follows. First, as the single-pass heterodimeric transmembrane receptor and/or in couple with Notch ligands on the cell surface, Notch1 mediates cell-to-cell interactions in adjacent cells and may therefore physically limits the migration of tumors cells [14]. Secondly, other lines of experimental evidence showed that without Notch1 activation, the adherens junctions complex containing E-cadherin is intact, which suppresses tumor cell migration and metastasis [22,23]. In NSCLC A549, NCI-H1650 and NCI-H596 cells, E-cadherin expression was decreased after transfection with a N1-ICD vector [24]. Notch1 down-regulated E-cadherin through upregulation of the snail family of transcriptional factors in these cell lines [24]. The reduction in E-cadherin by EDTA treatment can be somewhat rescued by RO4929097 in NCI-H358 and NCI-H322M cells. The data suggest that Notch1 activation is in part responsible for reducing E-cadherin. By contrast, activated Notch1 is not negatively associated with nodal metastasis. Rather, heterogeneous N1-ICD-V1754 expression is associated with heterogeneous tumor cell proliferation in NSCLC. In agreement with our results, a study found that Notch1 expression was inversely correlated with stage, despite lack of correlative data on nodal metastasis, in 395 NSCLC tumor samples by immunohistochemistry using a semi-quantitative scoring method [25]. Moreover, Notch1 expression predicted not only less progressive disease but also better overall survival in lung adenocarcinoma patients [26] Taken together, Notch1 plays distinct roles depending on its activation status in patients with all stages of NSCLC from I to IV.

Interestingly in this study, we found that Notch1and N1-ICD-V1754 are expressed in undifferentiated carcinomas of the lung, which has not been described previously [27]. Notch1and N1-ICD-V1754 were also found highly expressed in a case of undifferentiated carcinoma of the ovary [28]. Undifferentiated carcinoma is an epithelial malignancy that lacks morphologic or functional indicators of its embryonic origin, capable of deriving from many organ sites including bladder, cervix, colon, esophagus, larynx, pancreas, salivary glands, thyroid and uterine, besides lung and ovary [29,30]. Given the implication of the Notch pathway signaling in early development, Notch signaling may be of significance in the pathogenesis of undifferentiated carcinoma. Additional studies should investigate the role of Notch1 signaling in the pathogenesis of undifferentiated carcinomas.

## Conclusions

Expression of membranous and cytoplasmic Notch1expression is common, in contrast to the activated Notch1 in the nucleus, in human NSCLC [9,10]. Such pattern of expression or inactivated Notch1 may serve as a marker of low-metastatic propensity while high levels of or activated Notch1 a more pertinent therapeutic target in NSCLC or advanced NSCLC. We anticipate clinical validation of Notch1 and N1-ICD-V1754 expression levels and patterns for the purpose of evaluation of efficacy, ultimately patient stratification, and as a pharmacodynamics biomarker to document inhibition of Notch cleavage after treatment with the Notch pathway inhibitors in clinical trials.

## Methods

### Cells, cell culture, and EDTA and GSI treatment

The NSCLC cell lines NCI-H23 (adenocarcinoma), NCI-H522 (adenocarcinoma), NCI-H322M (adenocarcinoma) and NCI-H358 (adenocarcinoma) were obtained from the Tumor/Cell Line Repository, Division of Cancer Treatment and Diagnosis, National Cancer Institute. MCF-7 breast cancer cells were obtained from ATCC (Rockville, MD). Exponentially growing cells in 10% fetal bovine serum RPMI media at ~ 80% confluence were washed with serum-free RPMI. After washing, cells were treated with EDTA at a concentration of 0.53 mM or serum-free RPMI for 15 minutes and subsequently subjected to either formalin fixation and paraffin-embedding or cell lysis for lysates collection. Additionally, NCI-H358 and NCI-H322M cells were treated with 5 μM of a GSI RO4929097 (Selleck Chemicals, Boston, MA), for 10 min before adding EDTA solution containing 5 μM of RO4929097. After 15 min, lysates were collected or cells were fixed with 10% neutral buffered formalin before paraffin embedding.

### Western blot

Equal numbers of cells were lysed in Laemmli sample buffer and proteins in the lysates were separated by electrophoresis [31]. After protein separation and transferring, nitrocellulose filters were probed with monoclonal antibodies to Notch1 (clone EP1238Y, Abcam Inc., Cambridge, MA) and cleaved Notch1, N1-ICD-V1754 (clone D3B8, Cell Signaling Technology Inc., Danvers, MA) in dilutions of 1 to 5000 and 1 to 500, respectively. The Notch1 antibody recognizes the cytoplasmic portion of human Notch1 receptor. The cleaved Notch1 antibody specifically recognizes an epitope of Notch1 intracellular domain when cleaved at the protein sequence between G1753 and V1754 for human Notch1 (N1-ICD-V1754) or G1743 and V1744 for mouse and rat [16,32]. E-cadherin was detected by a monoclonal antibody against E-cadherin (clone HECD-1, Invitrogen Corp., Camarillo, CA) in 1 to 500 dilution and incubated for 1 hour at 37°C. ß-actin was probed with mAb at a dilution of 1: 10,000 as loading control (Sigma, St. Louis, MI). Reactive proteins were revealed using an enhanced chemiluminescence (Pierce Chemical Co., Rockford, Illinois, USA).

### Human lung cancer tissue microarray specimens

Lung cancer tissue microarrays and clinicopathological data were obtained from Cybrdi, Incorporation (Rockville, MD), and FOLIO Biosciences (Powell, OH). Approval of the biomarker study on de-identified human tissues was obtained from the Office of Human Research Protections, National Institutes of Health, Bethesda, Maryland. Tumor presence and histology were confirmed on HE stained sections.

### Immunohistochemistry or immunocytochemistry and quantitative analysis

Immunohistochemistry method on formalin-fixed and paraffin-embedded sections has been described previously [33,34]. In brief, after incubation of the primary antibodies to Notch1 and cleaved Notch1 in dilutions of 1: 200 and 1: 20 or antibody to E-cadherin in 1:100 dilution or antibody to Ki67 in 1: 200 dilution (clone MIB-1, DAKO) for 1 hour, binding of the antibodies to their antigen binding sites in sections was amplified using Vectastain Elite avidin-biotin-peroxidase complex kits (Vector Laboratories, Burlingame, CA). The antigen-antibody reaction sites were visualized using 3,3-diaminobenzidine for 7 min and, subsequently, sections were counterstained with Mayer’s hematoxylin. Paraffin-embedded NCI-H23 cells or MCF-7 cells treated with and without EDTA described above were used as controls; negative controls were performed using isotype immunoglobulins appropriate to the primary antibodies used. Areas of tumor staining on each tissue core were analyzed with assistance of a digital imaging system (DAKO, Carpinteria, CA) by reporting intensity and percentage of staining. Staining Index (SI) for Notch1 or N1-ICD-V1754 was calculated as the percentage multiplied by the intensity of staining (after subtracting the tissue readout of the corresponding negative control) divided by 100 (SI = intensity x percentage/100) [34]. It was defined as negative if SIs were < 2, and positive if the SIs were ≥ 2. As for defining the levels of expression, it was considered as the low level if SIs were ≥ 2 and <15 (1+ by manual scoring of intensity), as the moderate level if SIs were ≥ 15 and < 30 (2+), and as the high level if SIs were ≥ 30 (3+).

### Statistical analysis

The correlation between Notch1 or N1-ICD-1754 and age was analyzed using the Pearson’s correlation coefficient r statistic. All other correlations between Notch1 or N1-ICD-1754 and sex, grade, tumor size, nodal status or clinical stage were assessed by the Spearman’s rank order correlation coefficient. The two-sided *P*-values were calculated by means of Fisher transformation, and *P* values < 0.05 were considered statistically significant. Comparison of Notch1 percent positivity between squamous and adenocarcinoma was assessed by Fisher’s Exact test, two-sided *P*-value.

## Abbreviations

CI: Blyth-Still-Casella confidence interval
EDTA: Ethylenediaminetetraacetic acid
EGF-R: Epidermal growth factor receptor (EGF-R)
GSI: Gamma-secretase inhibitor
mAb: Monoclonal antibody
N1-ICD: Notch1 intracellular domain
NICD: Notch intracellular domain
NSCLC: Non-small cell lung cancer
